# Predictive Value of Serial ECGs in Patients with Suspected Myocardial Infarction

**DOI:** 10.3390/jcm9072303

**Published:** 2020-07-20

**Authors:** Jonas Lehmacher, Johannes Tobias Neumann, Nils Arne Sörensen, Alina Goßling, Paul Michael Haller, Tau Sarra Hartikainen, Peter Clemmensen, Tanja Zeller, Stefan Blankenberg, Dirk Westermann

**Affiliations:** 1 Department of Cardiology, University Heart and Vascular Center Hamburg, 20251 Hamburg, Germany; j.neumann@uke.de (J.T.N.); n.soerensen@uke.de (N.A.S.); a.gossling@uke.de (A.G.); p.haller@uke.de (P.M.H.); t.hartikainen@uke.de (T.S.H.); p.clemmensen@uke.de (P.C.); t.zeller@uke.de (T.Z.); s.blankenberg@uke.de (S.B.); d.westermann@uke.de (D.W.); 2 Deutsches Zentrum für Herz-Kreislauf-Forschung (DZHK), 10785 Berlin, Germany; 3 Department of Epidemiology and Preventative Medicine, Monash University, Melbourne VIC 3004, Australia; 4 Faculty of Health Sciences, Institute of Regional Health Research, University of Southern Denmark, Odense and Nykoebing F Hospital, 5230 Odense M, Denmark

**Keywords:** myocardial infarction, acute coronary syndrome, electrocardiography, outcome

## Abstract

The electrocardiogram (ECG) is an important diagnostic tool for patients with suspected acute myocardial infarction (AMI). Current guidelines recommend serial ECGs in case of persisting symptoms. We aimed to analyze the predictive value of ischemic ECG-signs in patients with suspected AMI. Patients presenting to the emergency department with suspected AMI were included. All patients with ST-elevation AMI were excluded from analyses. Patients received 12-lead-ECG and high-sensitive Troponin T (hs-TnT)-measurement at admission and after 3 h. Four groups were defined: no ischemic signs in either ECG; new ischemic signs in the second ECG; resolved ischemic signs in the second ECG; and persistent ischemic signs in both ECGs. Patients were followed for 2 years to assess the composite endpoint of all-cause-mortality, AMI, and coronary revascularization. Using a 30-day landmark analysis, a Cox regression with ischemic signs as the variable of interest, adjusted by cardiovascular risk factors, was calculated. Of 1675 patients, 1321 showed no ischemic signs, in 25 new-, in 92 resolved- and in 237 patients, persistent ischemic signs were documented. Patients with persistent ischemic signs had significantly worse outcomes, compared to those without. Compared to no ischemic signs, adjusted hazard ratios for the combined endpoint were 0.81 (95% CI 0.20, 3.31; *p*-value = 0.77) for new-, 0.59 (95% CI 0.26, 1.34; *p*-value = 0.21) for resolved-, and 1.47 (95% CI 1.102, 2.13; *p*-value = 0.041) for persistent ischemic signs. In patients with suspected AMI, persistent ischemic ECG-signs are predictive of a higher rate of all-cause-mortality, AMI, and revascularization.

## 1. Introduction

The electrocardiogram (ECG) is a pivotal part of the diagnostic workup of patients with suspected acute myocardial infarction (AMI), being the first diagnostic test provided in an acute setting. Current guidelines recommend documentation and interpretation of an ECG within the first ten minutes after presentation to an emergency department [1,2]. The admission ECG, as a fast and non-invasive diagnostic tool, provides important prognostic and diagnostic information, not only for diagnosis of ST-elevation myocardial infarction (STEMI) but also for non-ST-elevation myocardial infarction (NSTEMI) [3,4,5,6,7]. It is well established that ST-segment changes, especially ST-segment deviation (STD), are associated with poor outcomes [8,9,10,11]. Also, the amplitude of STD, as well as the number of leads showing STD, are known to be associated with a higher rate of adverse events [10,12,13]. These aspects already find consideration in several validated models for risk stratification [14,15,16,17]. For T-wave-inversion (TWI), previous study results have indicated that isolated TWI is not associated with poor outcomes, but occurring with STD simultaneously, they seem to be an independent indicator for higher risk of adverse events [4,5,6,9,18]. In cases of persistent symptoms or inconclusive diagnostic findings, serial ECGs are recommended (1). However, the optimal timing of obtaining a serial ECG is uncertain (1). Existing data on obtaining serial ECGs indicates that there may be an incremental prognostic value in persistent or dynamic changes in a second ECG [19,20,21,22,23]. Earlier studies have investigated the prognostic value of follow-up or discharge ECGs and showed that additional information can be derived from these ECGs [20,23]. Not all studies showed additional information for risk stratification and outcome besides those derived from admission ECG [19,21]. All the mentioned studies used different timepoints for documentation of the second ECG (at least 12 h after admission) [19,20,21,23]. Little is known about possible prognostic value of short-term serial ECGs in the emergency department in the first hours after presentation. Some studies regarding prognostic value of continuous ST-monitoring have shown that transient ischemic episodes predict a higher rate of short- and long term adverse cardiac events in patients with AMI [24,25,26]. In this study, we aimed to evaluate the predictive and prognostic value of ischemic signs and dynamic changes in short-term serial ECGs in patients with suspected AMI presenting to the emergency department.

## 2. Methods

We included patients of the Biomarkers in Acute Cardiac Care (BACC) cohort presenting with acute chest pain or other cardinal symptoms of acute coronary syndromes between 2013 and 2018 at the emergency department of the University Hospital Hamburg-Eppendorf (Hamburg, Germany). The inclusion criteria and the study protocol have been described in detail in previous publications [27,28,29]. Each patient was over 18 years old and provided written informed consent. For patients presenting multiple times during the inclusion period, only first presentation was included in the data set. The study is registered at clinicaltrials.gov (NCT02355457) and was approved by the local ethics committee Hamburg (PV4306) on 25 July 2013. The study was conducted in accordance with the Declaration of Helsinki and Good Clinical Practice.

Patients received an ECG and high-sensitive troponin T (hs-TnT, Elecsys^®^ troponin T high-sensitive; Roche Diagnostics) measurement at admission and after 3 h according to the 0/3 h algorithm of the European Society of Cardiology [2,30]. Additional patient characteristics (sex, age, pre-existing cardiovascular conditions, cardiovascular risk factors, body-mass index, and renal function (calculated using the Chronic Kidney Disease Epidemiology Collaborations’ equation)) were documented [31].

The ECGs were recorded as standard 12-lead-ECGs at a paper speed of 25 mm/s and calibrated for 1 mV/cm. The ECG was considered ischemic if one or more of the following findings were documented: significant ST-elevation (ST-elevation at the J point in 2 contiguous leads ≥ 0.1 mV, except V2 and V3 were sex-specific cut-offs apply) [7,32]; ST-deviation (horizontal or down-sloping STD ≥ 0.05 mV in 2 contiguous leads); TWI ≥ 0.1 mV in 2 contiguous leads with prominent R-wave or R/S ratio ≥ 1 (4); ventricular arrhythmias; and left or right bundle branch block or > 1st degree atrioventricular block. Based on the ECG results, four groups were defined as follows: no ischemic signs in either ECG; new ischemic signs in ECG after 3 h; resolved ischemic signs in ECG after 3 h; and persistent ischemic signs in both ECGs. During the stay at the emergency department, all ECGs were interpreted by the attending cardiologists in charge.

Final diagnosis was adjudicated in blinded fashion by two cardiologists considering all available clinical, laboratory, and imaging data, according to Third Universal Definition of Myocardial Infarction [7]. Diagnoses were categorized as AMI (divided in NSTEMI and STEMI), unstable angina pectoris, stable angina pectoris, cardiac non-coronary chest pain, and non-coronary chest pain. In this process, each obtained ECG also was reinterpreted by each of the two cardiologists. In cases of incongruent diagnosis or ECG interpretation, a third cardiologist was consulted. 

All patients were followed up with for at least 2 years via structured telephone interviews. In cases where telephone contact was repeatedly unsuccessful, a short questionnaire was sent, or the registration office was contacted. We defined a composite endpoint including either all-cause mortality, incident AMI, and revascularization (percutaneous coronary intervention (PCI) or coronary artery bypass graft). Any of these components was sufficient to count as an event. Events were only added to the composite endpoint if occurring after discharge from index hospitalization. For the present project, the endpoint was evaluated using a 30-day-landmark analysis, excluding all events which occurred within the first 30 days after index presentation. Continuous variables were shown as median (25th percentile, 75th percentile) and binary ones were described by relative frequencies (absolute). For relative frequencies, one decimal place was given. The above-mentioned combined endpoint Cox regression was calculated using ischemic signs as a variable of interest with the group of no ischemic signs as a reference. In addition to the unadjusted model, an adjusted Cox model with, age, sex, hypertension, diabetes, smoking, hyperlipoproteinemia, previously known congestive heart failure, as well as history of coronary artery disease (CAD), bypass or PCI as predictors, was used. Survival curves for a 2-year follow-up period after the 30-day landmark were produced using the Kaplan-Meier method and log-rank test was used to test for survival curve differences. The combined endpoint was the endpoint considered. Statistical significance was defined as *p*-value < 0.05. All statistical analyses were performed using R-Statistics Version 3.5.2 (R Foundation for Statistical Computing) [33].

## 3. Results

**Figure 1:**
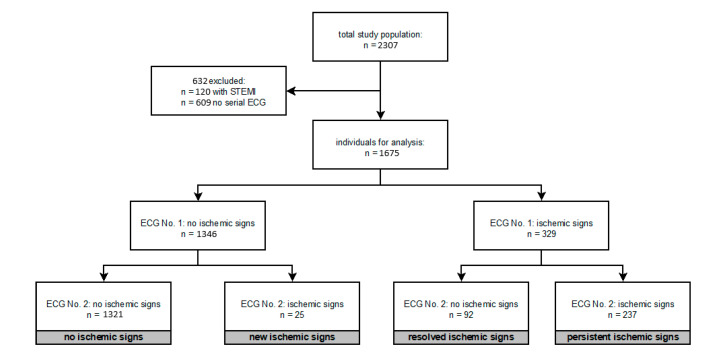
Overview of the study population.

In total we enrolled 2307 individuals. Excluding 632 individuals (120 STEMI, 609 with missing ECG information), 1675 patients remained for analysis. Divided into the above-mentioned four groups, 1321 (78.9%) patients did not show any ischemic signs in the ECG, 25 (1.5%) patients showed new ischemic signs in the ECG after 3 h, 92 (5.5%) patients had ischemic signs in the admission ECG which were resolved in the second ECG, and in 237 (14.1%) patients ischemic signs were documented in both performed ECGs (Figure 1).

Overview of the study population and subgroups of different ECG findings. Ischemic ECG signs defined as significant ST-elevation, ST-deviation, T-wave inversion ≥0.1 mV in 2 contiguous leads, ventricular arrhythmias; left or right bundle branch block or >1st degree atrioventricular block ECG = electrocardiogram; STEMI = ST-segment elevation myocardial infarction; ECG No. 1 = ECG obtained at presentation; ECG No. 2 = ECG obtained after three hours

On average, patients included were 65 years old (52; 75) and 64.1% (1073) were male (Table 1). Of all patients enrolled, 67.0% (1119) had prevalent hypertension, 36.1% (604) had previously known hyperlipoproteinemia, 12.6% (209) had diabetes mellitus, and 47.6% (794) were active or former smokers. A total of 34.4% (576) had a history of previously known coronary artery disease, underwent PCI or coronary artery bypass grafting. 6.6% (111) had previously suffered a stroke and in 11.7% (196) congestive heart failure had been diagnosed before. AMI was diagnosed in 15.6% (262) of the study group, 23.7% (397) underwent coronary angiography, of whom 46.8% (183) received PCI. The median time between the two obtained ECGs were 3 h and 24 min.

Patients with persistent ischemic signs were more likely to have hypertension (80.9% vs. 64.1% [resolved ischemic signs]/64.0% [new ischemic signs]/64.7% [no ischemic signs]) as well as diabetes (19.9% vs. 14.1/8.0/11.2%) than other patients. Also, in patients with persistent ischemic signs in both ECGs, a history of coronary artery disease, PCI or coronary artery bypass grafting was documented more often (49.4% vs. 39.1/36.0/31.3%), as well as a higher percentage having already suffered AMI (28.0% vs. 10.9/8.0/14.9%). Additionally, congestive heart failure had been previously diagnosed in those patients more frequently (24.9% vs. 7.6/4.0/9.8%). Patients with persistent ischemic ECG findings also were diagnosed with AMI more frequently (36.3% vs. 23.9/16.0/11.4%), underwent coronary angiography more often (42.6% vs. 35.9/36.0/19.2%), as well as PCI (23.6% vs. 14.1/16.0/8.3%), than other patients.

We also compared baseline characteristics between included patients and those who were excluded due to missing data (Table S1). Of the two groups, excluded patients were more frequently active smokers, (28.1% vs. 21.6%; *p* = 0.0013) and were more likely to have previously known congestive heart failure (14.9% vs. 11.7%; *p* = 0.046). Also, excluded patients received angiography (42.2% vs. 23.7%; *p* < 0.001) and revascularization (30.2% vs. 10.9%; *p* < 0.001) more frequently. There were no significant differences considering other documented baseline characteristics. Regarding the final diagnosis, excluded patients had a significantly higher rate of AMI (36.1% vs. 15.6%; *p* < 0.001).

Of 329 individuals with ischemic signs in the admission ECG, 50.2 % (165) showed TWI, 40.1% (132) STD, and 7.9% (26) ST-segment elevation (Table 2). The most frequent ischemic sign in the group with resolved ischemic ECG was STD, whereas TWI was the most common sign in patients with persistent ischemic ECG. In the group of new ischemic ECG signs (*n* = 25) TWI was also the most common finding with a frequency of 64% (16). Only six patients in the study population (five in the group of persistent ischemic signs and ones in the group of resolved ischemic signs) showed other ECG findings (bundle branch block, ventricular arrythmia, or atrioventricular block >1st degree).

Follow-up information was available for 1673 patients (99.9%). In the 2-year follow-up time after the 30-day landmark, 172 events occurred. The unadjusted HR for the combined endpoint showed no significant difference between the group with no ischemic ECG signs and the groups of new- (HR 0.80; 95% CI 0.20, 3.24; *p* = 0.76) and resolved ischemic signs (HR 0.68; 95% CI 0.30, 1.55; *p* = 0.36). The group with persistent ischemic ECG signs showed significantly higher HR than the reference group (HR 2.09; 95% CI 1.47, 2.98; *p* < 0001) (Table 3).

After adjusting for age, sex, diabetes, smoking, hypertension, hyperlipoproteinemia, congestive heart failure, and history of CAD, bypass or PCI, the adjusted hazard ratio (aHR) for the combined endpoint is still significantly higher for the group with persistent ischemic signs in both ECGs (aHR 1.47; 95% CI 1.02, 2.13; *p* = 0.041) (Table 4). For the other two groups, HRs still did not significantly differ from the reference group. Of the factors adjusted for, age (HR 1.05; 95% CI 1.03, 1.06; *p* < 0.001), male sex (HR 1.65; 95% CI 1.13, 2.29; *p* = 0.0082), diabetes (HR 1.60; 95% CI 1.11, 2.29; *p* = 0.011) as well as congestive heart failure (HR 1.47; 95% CI 1.01, 2.12; *p* = 0.042), and history of CAD/Bypass/PCI (HR 1.44; 95% CI 1.01, 2.05; *p* = 0.042) showed significant influence on the composite endpoint, increasing the risk of death, AMI, and coronary revascularization during follow-up time.

**Figure 2:**
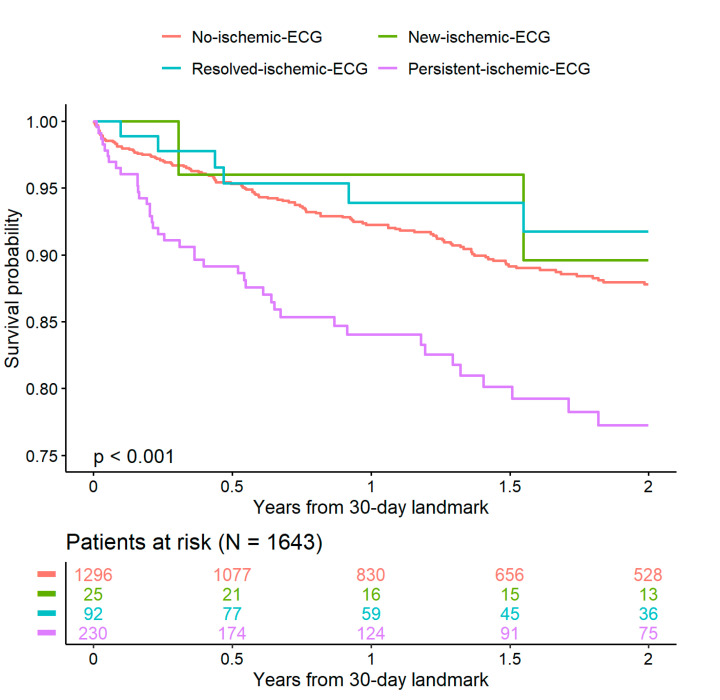
Survival curves for the combined endpoint according to ischemic groups. Figure 2 shows Table 4 subgroups (no-ischemic-ECG, new-ischemic-ECG, resolved-ischemic-ECG, persistent-ischemic ECG), from a 30-day landmark, taking only into account events occurring 30 days after index hospitalization.

Figure 2 shows the truncated survival curves of each of the four subgroups with the numbers of patients at risk below. Individuals with persistent ischemic ECG findings showed worse outcomes for the combined endpoint than the other groups.

## 4. Discussion

The key finding of this work is that in patients with suspected AMI persistent ischemic signs in a performed ECG immediately at presentation to an emergency department and after 3 h are predictive for a significantly worse outcome regarding a composite endpoint including all-cause-mortality, incident AMI, and coronary revascularization compared to those without ischemic ECG signs in either performed ECG. For patients with new- or resolved ischemic signs in the second ECG, no such difference could be found.

STD on the admission ECG is well known to indicate a poor prognosis in patients with suspected AMI [4,5,6,8,9,10,11,18]. Also, previous studies which have investigated the possible prognostic impact of dynamic ST-segment changes during follow-up or on the discharge ECG revealed mixed results [19,20,21,22,23]. The inclusion criteria of the cited studies partly differ substantially but can roughly be categorized in two groups. Krone et al. and Schechtman et al. only included patients with confirmed AMI [11,22]. The other mentioned works included patients with ACS (unstable angina pectoris or AMI) if patients met additional inclusion criteria like invasive diagnostic, ECG changes, or fulfilling the Global Registry of Acute Coronary Events (GRACE) criteria [4,5,6,8,19,20,21,23]. Though all studies aimed to analyze the value of ECG changes (TWI or STD) as an independent prognostic predictor [4,5,6,8,9,10,11,18,19,20,21,22,23]. As the main finding of the present study, persistent ischemic signs, even in a short-term serial ECGs, are associated with a significantly worse long-term outcome and higher risk for AMI, revascularization, or death in patients with suspected AMI. For patients with new or resolved ischemic ECG signs, we did not find any significant prognostic influence compared to patients without any ischemic signs. However, the number of cases with new- and resolved ischemic ECG signs was rather small in our study population, which might result in under- or overestimation of their prognostic value. Nevertheless, these results are in line with those of Hersi et al., who showed that persistent STD on the discharge ECG are associated with a higher rate of AMI or death within 6 months. Also, Yan et al. showed that persistent STD, as well as the magnitude of STD in a follow-up ECG within 12–24 h after admission, predicts a higher rate of death or AMI within 6 months [23]. Yan et al. also adjusted for the status of STD on the admission ECG and showed a higher unadjusted mortality for STD on follow-up ECGs independently of their presence on the admission ECG [23]. In our study, we did not explicitly stratify for STD or ischemic signs in the admission ECG. Yet our analyses indicate an incremental prognostic value of persisting ischemic ECG findings, as there was no significant difference in outcome between patients without any ischemic signs or those with resolved ischemic signs after 3 h, whereas persistence of ischemic signs was clearly associated with higher rate of events during follow-up. Alkaabi et al. analyzed the possible prognostic value of STD in admission and discharge ECG (performed 24 to 36 h after admission). Their work found no incremental prognostic value adding to the ECG performed on admission. In that study, patients with documented STD in general had a significantly worse outcome than those without [19]. In contrast, these findings were not observed in our study. Sarak et al. investigated the possible prognostic value predicting mortality in patients with TWI in consecutive ECGs (performed on admission and between 24 to 48 h after). The group did not find any incremental prognostic value regarding this type of ischemic ECG sign [21].

In current ESC NSTEMI guidelines, dynamic ECG changes, especially ST- or T-wave changes, are an indicator for high ischemic risk, mandating invasive diagnostic and/or therapy in patients presenting with suspected non-ST-segment-elevation-acute coronary syndrome [2]. As acute risk assessment was not the focus of our work, our findings do not generally affect this statement, as we focused on long term prognosis and therefore portability is limited.

Our study shows several differences with previous studies investigating the prognostic value of serial ECG writing. First, by repeating the ECG after 3 h, we chose a completely different time of writing the second ECG than the cited studies, which wrote follow-up ECGs at least 12 h after the initial one [19,20,21,22,23]. In those studies, in a varying frequency, follow-up ECGs were performed after coronary angiography or revascularization, so an influence of unknown relevance on possible ECG changes has to be assumed and could not be excluded [20,22,23]. In our study, this influence is reduced to a minimum due to the short period between first and second ECG (median 3 h and 24 min). Patients who underwent urgent revascularization during the first hours after presentation (for example, patients presenting with STEMI) almost completely lacked serial ECG information and consequently were excluded from analyses. Regarding the baseline characteristics of excluded patients, except for smoking status and previously known congestive heart failure, we did not find significant differences regarding the cardiovascular risk profile. However, a significantly greater proportion of excluded patients suffered AMI and therefore have a higher risk for cardiovascular events and death after index hospitalization per se [2,34]. This finding is likely explained by a selection bias, as patients with AMI will undergo further treatment more quickly and thus receive a second ECG less often.

Second, we did not focus on one single kind of ischemic ECG sign like STD or TWI, as was the case in the several previous studies, but looked at ischemic ECG changes as a whole [19,20,21,22,23]. Since we did not separately analyze the prognostic value of STD, TWI, or other isolated ischemic signs, we cannot state whether one specific ECG finding has a stronger predictive value than others. Yet, results of several previous studies indicate that TWI is not as predictive for poor prognosis than STD, so it may be assumed that the predictive value of persistent ischemic ECG signs is mainly driven by the occurrence of STD in serial ECGs which, in our study, was the case for 33.5% in that specific subgroup [4,5,6,9,18,21]. On the other hand, the main ECG finding in this group has been TWI (56.8%), so if persistent STD would be the main negative predictor, the real effect could be much more distinct than we observed.

Third, our follow-up period of 2 years after a 30-day landmark was much longer compared to previous studies, with follow-up periods of up to 1 year maximum [19,20,21,22,23]. In addition, our follow-up was nearly complete with available follow-up information for 99.9% of our cohort. Therefore, our results add additional information on prognosis of patients with persistent ischemic ECG, even beyond a time period of 1 year.

Fourth, using a combined endpoint considering all-cause-mortality, incident AMI, and myocardial revascularization adds additional information about relevant and most certainly symptomatic coronary artery disease being treated before leading to AMI. However, it cannot be excluded that this component may be self-driven, because it is rather likely that persistent ischemic ECG changes, even if acute ischemia is ruled out, lead to further stress-testing and consequently to a higher rate of revascularization than, e.g., patients without pathological ECG findings.

Our results suggest that even if persisting over a short time, ischemic ECG signs already indicate a higher risk of death, AMI, or revascularization. While our results may not be compared directly, these findings are corroborated by studies evaluating the prognostic value of continuous 12-lead ECG monitoring in patients with AMI [24,25,26]. These studies showed that transient ischemic episodes are predictive for early, as well as long-term adverse cardiac events [24,25,26]. Sensitivity of automated ST-monitoring was shown to vary dependent on the chosen threshold for significant ST-shifts [35]. However, automated continuous ST-monitoring may identify patients with transient myocardial ischemia, who would have been missed due to under-sampling or human interpretation [36,37]. As far as we know, current algorithms do not consider TWI as a possible ischemic sign. Hence, continuous 12-lead ECG-monitoring is not widely available and not well established in clinical routine, and serial ECG writing in short time-periods could also possibly detect transient ischemia as a predictor for adverse events as a bridge until cost-effectiveness analyses are accessible and system availability increases. With 1675 individuals for analyses, our sample size is rather large compared to those of Hersi et al. (918 patients) and Schechtman et al. (515 patients), but far exceeded by the cohort from Global Register of Acute Coronary Events and Acute Coronary Syndrome Registry in Sarak et al.’s study (7201 patients) [20,21,22]. Regarding the baseline characteristics of comparable studies, our sample fits in with mostly male patients of higher age and a high prevalence of cardiovascular risk factors and pre-existing cardiovascular conditions [19,20,21,22,23]. However, there are minor variations between all mentioned samples, assumingly due to different patient selection [19,20,21,22,23].

Aside from already examined ECG parameters like STD and TWI, regarding their role in acute or long-term risk stratification in suspected or confirmed AMI, there are some open issues in ECG analysis which remain unclear. For example, the diagnostic and prognostic values of *p*-wave abnormalities detecting atrial ischemia and risk for supraventricular arrhythmia are still elusive, as well as the prognostic value of Q-wave development after treatment of AMI [38]. Furthermore, the value of ECG analysis to identify the culprit lesion or acute coronary occlusion and prognostic value of certain T-wave morphologies all need further clinical investigation to assess their potential clinical applicability [38]. Gragnano et al. addressed these issues in a recent work, presenting the ECG-MATRIX sub-study, designed to analyze the clinical value of numerous ECG parameters in more than 4000 ACS patients [38].

Our study has some limitations. First, it is a single-center study, so despite its rather large sample size, generalizability of the results is potentially limited. Second, enrollment of patients was not 24 h a day, due to limited personnel capacity, resulting in an inherent selection bias focusing only on patients presenting at daytime. Third, the inclusion of any patients with suspected AMI without further inclusion criteria regarding certain ECG findings resulted in a rather small percentage of patients with ischemic ECG signs, which is partly compensated by the large sample size. This problem occurred not only in our study, but also in the investigation of Hersi et al. and Alkaabi et al. [19,20]. On the other hand, the rather unselected inclusion of patients makes our results more applicable to clinical routine than a more strictly selected sample. Fourth, as we did not have access to ECGs obtained before initial presentation at the emergency department, it is possible that persistent ischemic ECG signs are a result of pre-existing ischemic heart disease or congestive heart failure, which would be predictors for impaired prognosis themselves according to current guidelines [2]. Yet, after adjusting for congestive heart failure, as well as for history of CAD, bypass or PCI, persistent ischemic signs are still an independent predictor for risk of all-cause-mortality, AMI, and revascularization during the follow-up period. Fifth, the results of the ECGs were not blinded to the physicians in the emergency department, so that it cannot be assured that persistent or evolving dynamic ECG changes did not lead to therapy intensification resulting in a possible better long-term outcome.

## 5. Conclusions

Persistent ischemic signs in short-term serial ECGs predict a significantly higher long-term risk for death, AMI, and myocardial revascularization in patients with suspected AMI and therefore could help identify patients who may benefit from intensified therapy.

## Figures and Tables

**Table 1:** Baseline characteristics for study population and each subgroup.

	All (*n* = 1675)	No-Ischemic-Signs (*n* = 1321)	New-Ischemic-Signs (*n* = 25)	Resolved-Ischemic-Signs (*n* = 92)	Persistent-Ischemic-Signs (*n* = 237)
Age (years)	65.0 (52.0, 75.0)	63.0 (51.0, 74.0)	67.0 (53.3, 76.0)	69.0 (55.4, 77.0)	71.0 (58.0, 78.0)
Male No. (%)	1073 (64.1)	858 (65.0)	16 (64.0)	59 (64.1)	140 (59.1)
Hypertension No. (%)	1119 (67.0)	853 (64.7)	16 (64.0)	59 (64.1)	191 (80.9)
Hyperlipoproteinemia No. (%)	604 (36.1)	462 (35.0)	10 (40.0)	28 (30.4)	104 (43.9)
Diabetes No. (%)	209 (12.6)	147 (11.2)	2 (8.0)	13 (14.1)	47 (19.9)
Current smoker No. (%)	360 (21.6)	291 (22.1)	5 (20.0)	15 (16.3)	49 (20.9)
Former smoker No. (%)	434 (26.0)	352 (26.7)	5 (20.0)	22 (23.9)	55 (23.4)
History of CAD/Bypass/PCI No. (%)	576 (34.4)	414 (31.3)	9 (36.0)	36 (39.1)	117 (49.4)
History of AMI No. (%)	275 (16.4)	197 (14.9)	2 (8.0)	10 (10.9)	66 (28.0)
Stroke No. (%)	111 (6.6)	85 (6.4)	2 (8.3)	2 (2.2)	22 (9.3)
Congestive heart failure No. (%)	196 (11.7)	129 (9.8)	1 (4.0)	7 (7.6)	59 (24.9)
BMI (kg/m^2^)	26.1 (23.7, 29.5)	26.0 (23.5, 29.4)	26.2 (22.8, 30.6)	27.0 (24.4, 29.2)	26.2 (23.8, 30.0)
eGFR (mL/min for 1.73m^2^)	76.7 (58.2, 92.4)	78.8 (61.1, 93.8)	80.1 (47.4, 89.9)	77.0 (52.4, 88.4)	64.0 (46.9, 82.1)
AMI No. (%)	262 (15.6)	150 (11.4)	4 (16.0)	22 (23.9)	86 (36.3)
Angiography No. (%)	397 (23.7)	254 (19.2)	9 (36.0)	33 (35.9)	101 (42.6)
PCI No. (%)	183 (10.9)	110 (8.3)	4 (16.0)	13 (14.1)	56 (23.6)
Time between ECG #1 and ECG #2 (h)	3.4 (3.1, 3.7)	3.4 (3.1, 3.7)	3.3 (3.1, 3.7)	3.4 (3.0, 3.7)	3.4 (3.1, 3.7)

CAD = coronary artery disease; PCI = percutaneous coronary intervention; AMI = acute myocardial infarction; BMI = body mass index; eGFR = estimated glomerular filtration rate; ECG = electrocardiogram; ECG #1 = ECG at presentation; ECG #2 = ECG after 3 h.

**Table 2:** Type of ischemic ECG findings for study population and each subgroup.

	All (*n* = 1675)	No-Ischemic Signs (*n* = 1321)	New-Ischemic Signs (*n* = 25)	Resolved-Ischemic Signs (*n* = 92)	Persistent-Ischemic Signs (*n* = 237)
ST segment depression ECG #1 No. (%)	132 (7.9)	0 (0)	0 (0)	51 (55.4)	81 (34.3)
ST segment elevation ECG #1 No. (%)	26 (1.6)	0 (0)	0 (0)	7 (7.6)	19 (8.1)
T-wave-inversion ECG #1 No. (%)	165 (9.9)	0 (0)	0 (0)	33 (35.9)	132 (55.9)
ST segment depression ECG #2 No. (%)	86 (5.1)	0 (0)	7 (28.0)	0 (0)	79 (33.5)
ST segment elevation ECG #2 No. (%)	21 (1.3)	0 (0)	2 (8.0)	0 (0)	19 (8.1)
T-wave-inversion ECG #2 No. (%)	150 (9.0)	0 (0)	16 (64.0)	0 (0)	134 (56.8)

ECG = electrocardiogram.

**Table 3:** Unadjusted hazard ratios (HR) for composite endpoint.

	HR (95% CI)	*p*-Value
New ischemic ECG	0.80 (0.20, 3.24)	0.76
Resolved ischemic ECG	0.68 (0.30, 1.55)	0.36
Persistent ischemic ECG	2.09 (1.47, 2.98)	<0.001

Hazard ratios for combined endpoint (all-cause mortality, acute myocardial infarction, and percutaneous coronary intervention) and “no ischemic electrocardiogram” as a reference group; HR = hazard ratio.

**Table 4:** Adjusted hazard ratios for composite endpoint.

	HR (95% CI)	*p*-Value
New ischemic ECG	0.81 (0.20, 3.31)	0.77
Resolved ischemic ECG	0.59 (0.26, 1.34)	0.21
Persistent ischemic ECG	1.47 (1.02, 2.13)	0.041
Adjusted for:		
Age, years	1.05 (1.03, 1.06)	<0.001
Male	1.61 (1.13, 2.29)	0.0082
Diabetes	1.60 (1.11, 2.29)	0.011
Ever smoker	1.13 (0.82, 1.56)	0.44
Hypertension	1.16 (0.74, 1.80)	0.52
Hyperlipoproteinemia,	0.96 (0.68, 1.34)	0.80
Congestive heart failure	1.47 (1.01, 2.12)	0.042
History of CAD/Bypass/PCI	1.44 (1.01, 2.05)	0.042

Hazard ratios for combined endpoint (all-cause mortality, acute myocardial infarction, and percutaneous coronary intervention) and “no ischemic electrocardiogram” as reference group; HR = hazard ratio; CI = confidence interval; CAD = coronary artery disease; PCI = percutaneous coronary intervention.

## Supplementary Materials

The following are available online at https://www.mdpi.com/2077-0383/9/7/2303/s1, Table S1: Baseline characteristics of included and excluded patients.
