# Supplementary material for: Predictive Value of Serial ECGs in Patients with Suspected Myocardial Infarction
Source: J Clin Med. 2020 Jul 20;9(7):2303. doi: 10.3390/jcm9072303 (PMC7408822; doi:10.3390/jcm9072303)
Supplement: Supplementary file 1 [file jcm-09-02303-s001.pdf]

Table S1. Baseline characteristics of included and excluded patients.

	**All (*N* = 2307)**	**Included (*N* = 1675)**	**Excluded Because of Missings in ECG (*N* = 609)**	**P-value Incl. vs Excl. ECG-missings**
Age (years)	65.0 (52.0, 75.0)	65.0 (52.0, 75.0)	65.0 (51.0, 74.0)	0.33
Male No. (%)	1481 (64.2)	1073 (64.1)	391 (64.2)	0.99
Hypertension No. (%)	1543 (67.1)	1119 (67.0)	409 (67.5)	0.85
Hyperlipoproteinemia No. (%)	827 (35.8)	604 (36.1)	218 (35.8)	0.95
Diabetes No. (%)	295 (12.9)	209 (12.6)	84 (14.0)	0.42
Current smoker No. (%)	537 (23.4)	360 (21.6)	170 (28.1)	0.0013
Former smoker No. (%)	578 (25.2)	434 (26.0)	142 (23.5)	0.25
History of CAD/Bypass/PCI No. (%)	775 (33.6)	576 (34.4)	195 (32.0)	0.31
History of AMI No. (%)	369 (16.0)	275 (16.4)	92 (15.1)	0.49
Stroke No. (%)	146 (6.3)	111 (6.6)	35 (5.7)	0.51
Congestive heart failure No. (%)	290 (12.6)	196 (11.7)	91 (14.9)	0.046
BMI (kg/m^2)	26.2 (23.7, 29.6)	26.1 (23.7, 29.5)	26.2 (23.6, 29.8)	0.62
eGFR (mL/min for 1.73m^2)	76.4 (58.4, 91.9)	76.7 (58.2, 92.4)	75.6 (58.7, 90.9)	0.75
AMI No. (%)	505 (21.9)	262 (15.6)	220 (36.1)	<0.001
Angiography No. (%)	676 (29.3)	397 (23.7)	257 (42.2)	<0.001
Revascularization No. (%)	387 (16.8)	183 (10.9)	184 (30.2)	<0.001
Time between ECG #1 an ECG #2 (h)	3.4 (3.1, 3.7)	3.4 (3.1, 3.7)	3.5 (3.1, 5.3)	0.49

CAD = coronary artery disease; PCI = percutaneous coronary intervention; AMI = acute myocardial infarction; BMI = body mass index; eGFR = estimated glomerular filtration rate; ECG = electrocardiogram; ECG #1 = ECG at presentation; ECG #2 = ECG after 3 hours.
